# Acceptability and Implementation Challenges of Benzathine Penicillin G Secondary Prophylaxis for Rheumatic Heart Disease in Ethiopia: A Qualitative Study

**DOI:** 10.5334/gh.1393

**Published:** 2025-01-29

**Authors:** Eshetie Melese Birru, Kevin T. Batty, Laurens Manning, Stephanie L. Enkel, Brioni R. Moore

**Affiliations:** 1 Curtin Medical School, Curtin University, Bentley, Western Australia, AU; 2 Department of Pharmacology, School of Pharmacy, College of Medicine and Health Sciences, University of Gondar, Gondar, Ethiopia; 3 Curtin Health Innovation Research Institute, Curtin University, Bentley, Western Australia, AU; 4 Medical School, The University of Western Australia, Crawley, Western Australia, AU; 5 Wesfarmers Centre of Vaccines and Infectious Diseases, The Kids Research Institute Australia, Nedlands, Western Australia, AU

**Keywords:** Rheumatic heart disease, Benzathine Penicillin G, COM-B model, Theoretical framework of acceptability

## Abstract

**Background:**

Monthly intramuscular injections of benzathine penicillin G (BPG) remain the cornerstone of secondary prophylaxis for acute rheumatic fever and rheumatic heart disease (RHD). The barriers to successful delivery of BPG may be patient- or service-delivery-dependent.

**Objective:**

The aim of the present study was to explore the perceived acceptability and implementation challenges of BPG treatment for RHD, from the perspective of healthcare providers (HCPs).

**Methodology:**

A descriptive qualitative study using semi-structured interview guides was conducted in four public hospitals in Ethiopia. Physicians and nurses who had at least 1 year of experience in delivering RHD secondary prophylaxis were recruited. The interviews were audio recorded, transcribed verbatim, and translated into English for analysis using framework method thematic analysis. Identified behavioral factors were mapped onto a theoretical framework of acceptability (TFA), and the Capability, Opportunity, Motivation-Behavior (COM-B) model.

**Result:**

Twenty-two interviews were conducted with HCPs (mean age 39 years, 55% nurses). Insights into BPG use and acceptability were categorized into four major themes related to: (1) individual factors (e.g., fear of anaphylactic reaction), (2) health system barriers (e.g., BPG shortage), (3) patient/caregiver perceptions (e.g., reliance on injectables, over expectation of treatment outcomes), and (4) product (e.g., injection pain, needle blockage).

**Conclusions:**

HCPs identified facilitators and barriers which highlight the complexities associated with BPG as secondary prophylaxis for RHD in Ethiopia. Based on these data, we suggest RHD control programs should (1) provide cross-disciplinary training and education programs to support safe and context-appropriate delivery of BPG (2) improve resourcing of health facilities to facilitate safe drug delivery, (3) establish a comprehensive system for auditing severe adverse reactions post-BPG injection to generate robust pharmacovigilance data, and consider alternative approaches to BPG delivery including access to improved formulations (e.g., BPG suspension formulations in pre-filled syringes).

**Highlights:**

– Key barriers included (a) resistance from healthcare providers to administer benzathine penicillin G (BPG) due to their concerns about injection-related severe adverse events, and potential repercussions should an event occur, (b) poor community and healthcare provider awareness of the disease and its treatment, (c) lack of resources to manage adverse events, and (d) injection pain.
– Key enablers included (a) perceived superior treatment benefits of BPG and (b) co-administration of lidocaine/analgesics to reduce injection pain.
– Recommendations to address identified challenges include (a) improved training/education on RHD diagnosis, disease progression, and treatment, (b) improved access to supportive resources, (c) active adverse reaction monitoring and reporting, and (d) encouraging the provision/access of globally subsidized BPG suspension formulations in pre-filled syringes.

## Introduction

Acute rheumatic fever (ARF) is an illness following an autoimmune response to *Streptococcus pyogenes* (Group A Streptococcus) infection of the upper respiratory tract, skin or mucosa (1). If untreated, ARF can affect the heart valves leading to rheumatic heart disease (RHD) (2). This preventable complication remains a major public health problem. In 2019, the global incidence and prevalence of RHD were estimated at 2.8 million and 40.5 million respectively, resulting in >300,000 deaths and 10.7 million disability-adjusted life-years (3). Despite declines in global mortality- and disability-adjusted life-years trends worldwide, the burden of RHD remains concerningly high in low- and middle-income countries within Oceania, South Asia, and sub-Saharan Africa, and in underprivileged populations within developed nations; e.g., Aboriginal and immigrant populations within Australia (4,5,6).

Benzathine penicillin G (BPG) remains the recommended secondary prophylaxis to prevent recurrent Group A Streptococcal infections by the World Health Organization (WHO) and is the cornerstone of ARF/RHD control programs (7). While BPG prophylaxis is highly effective and affordable, suboptimal delivery of BPG (7) is a common challenge in many endemic nations, including Ethiopia (8,9,10), resulting in increased morbidity and progressive heart damage (11).

The burden of rheumatic heart disease is high in Ethiopia, with a prevalence of approximately 3% (12). When diagnosed, most patients already have established severe valvular damage (13). The national treatment guideline recommends monthly intramuscular BPG injections for secondary prophylaxis of ARF/RHD (14). However, only two-thirds of RHD patients receive optimal BPG secondary prophylaxis, defined as receiving at least 80% of planned injections (8,15).

In most low- and middle-income countries, the only injectable BPG formulation available is a powdered preparation. Compared with more expensive suspensions in pre-filled syringes available in other settings (e.g. Bicillin^®^), powdered BPG preparations have reported quality concerns which may contribute to needle blockage and pharmacokinetic variability (16,17). Moreover, studies from Ethiopia (18), Uganda (19), and Sudan (20) have demonstrated low knowledge and skills of service providers relevant to ARF/RHD as barriers to successful control programs (21,22).

Anecdotal evidence suggests that suboptimal adherence to BPG injections may be due to fear of needle blockages and severe adverse events, which are sometimes fatal (23,24). Although the actual risk of anaphylaxis due to penicillin is low, occurring in 1 in 100,000 individuals (25), a survey by the World Heart Federation found that among 39 clinicians prescribing BPG, 20% reported a patient dying from anaphylaxis, while 26% were aware of a fatal anaphylactic reaction to BPG (24). As a result, healthcare providers (HCPs) may prescribe oral antimicrobials such as amoxicillin or phenoxymethylpenicillin, which are less effective alternative therapies for ARF/RHD (16,26,27,28,29).

Despite emerging evidence supporting subcutaneous delivery (30,31,32), RHD secondary prophylaxis currently requires painful monthly intramuscular injections over prolonged periods, leading to poor treatment uptake (9,33,34,35). Addressing the low uptake of secondary prophylaxis for ARF/RHD has been identified as a public health priority (26,36). Health sector-level barriers to BPG delivery are incompletely understood, with limited published research on the perspectives of HCPs and other stakeholders who play a decisive role in the delivery of BPG (21,26,33). The aim of this study was, therefore, to gain insight into the drivers of BPG acceptability for secondary prophylaxis of ARF/RHD in Ethiopia, from the perspective of HCPs.

## Methods

A descriptive qualitative study design was implemented to explore the perspectives of HCPs on patient experience, treatment acceptability, and implementation challenges of BPG secondary prophylaxis for ARF/RHD. The Consolidated Criteria for Reporting Qualitative Research (COREQ) (37) was used to design, conduct and report study findings (Supplementary Table 1).

### Ethical Approval

Ethical approval was obtained from the University of Gondar Institutional Review Board (VP/RTT/05/756/2022) and Curtin University Human Research Ethics Committee (HRE2022–0221).

### Site selection

To identify varying practices of secondary prophylactic care across Ethiopia, four public hospitals were selected for recruitment of participants: Gondar (University of Gondar Hospital; Amhara Region), Debre Tabor (Debre Tabor Comprehensive Specialized Hospital; Amhara Region), Dire Dawa (Dil-Chora Referral Hospital, Dire Dawa City Administration), and Arba Minch (Arba Minch Comprehensive Specialized Hospital; South Region). The selected hospitals are the only secondary or tertiary care hospitals in their catchment area. As the Ethiopian health sector development plan has implemented a three-tiered health-delivery system, general hospitals provide secondary-level services to 1–1.5 million people, and specialized referral hospitals provide tertiary services to 3.5–5 million people (38).

### Participants

HCPs (physicians and nurses) with at least 1 year of experience in delivering RHD secondary prophylactic care services and willing to participate in the study and provide informed consent, were recruited using purposive sampling methods. This enabled the recruitment of a diverse range of participants (age and duration of experience). The sample size was determined using the principles of thematic saturation (39). While data saturation was agreed to have been achieved after 18 interviews, due to altered treatment practice at one research site (Gondar), a further four participants were interviewed to ensure thematic consistency.

### Data collection

Semi-structured interview topic guides were developed, being informed by previous reports (40) and pilot observations of perceived gaps at one of the study sites (Gondar). Topic guides were piloted with two senior service providers (one nurse and one physician) to ensure questions were clear and comprehensive. Minor changes were made to the order and structure of the questionnaire. Semi-structured questionnaires (Supplementary text file) were prepared in both local (Amharic) and English languages, with participants able to select their language of choice.

At each study site, key staff involved in the delivery of ARF/RHD secondary prophylaxis were contacted with support letters from the University of Gondar to participate in the study and to identify other potential staff to be invited to participate. After introducing the study objectives written, informed consent was obtained prior to the interview and participants were guaranteed confidentiality to encourage full and free reflection of their views. In-depth interviews were conducted in person by a trained investigator (EMB, PhD student, Male, no prior relationship with participants) at each respective health institution, between April and June 2022. Each interview spanned 15 to 45 min and was audio-recorded.

### Data analysis

Audio-recorded interviews were transcribed verbatim, translated by Google Translate, and edited by an investigator fluent in Amharic and English (EMB). To ensure accuracy and reliability, a randomly selected subset of transcripts and translations were crosschecked against the audio records using back-translation (to local language) by an independent reviewer.

Using qualitative description analysis, transcribed responses were evaluated (EMB) to identify patterns, themes, commonalities, and differences, and to develop codes for study variables using QSR NVivo [Release 1.7.1 (1534), QSR International, Massachusetts, USA]. Similar codes were categorized into coherent and defined domains. To finalize the analysis of the shared perspectives between subjects, themes of responses were generated inductively using the framework method thematic analysis (41). The emerging sub-themes of perceived barriers and facilitators for the BPG secondary prophylaxis implementation services were then mapped onto the Capability, Opportunity and Motivational-Behavior (COM-B) (42) (Supplementary Table 2) and Theoretical Framework of Acceptability (TFA) (43) (Supplementary Table 3) models, and potential solutions mapped onto the Behavioral Change Wheel (BCW) model (42) (Supplementary Table 4). The integration of the TFA, which assesses service/intervention acceptability (43), and the COM-B model, which identifies factors influencing behavior (42), offers a practical approach to pinpoint key areas for successful practice changes and insights into strategies to overcome challenges to evidence-based change. To ensure consistency of analysis, six (~25%) transcripts were randomly selected and reviewed by a second investigator (BRM) for theme selection and coding. All discrepancies were reviewed (EMB and BRM), and theme tags were refined and redistributed to reach a consensus. The sociodemographic data of participants were described by frequencies, percentages, or means ± SD, as appropriate.

## Results

A total of 22 participants were recruited into the study across all research sites: Arba Minch (*n* = 4), Debre Tabor (*n* = 6), Dire Dawa (*n* = 4), and Gondar (*n* = 8). Of these, 10 participants (45%) were female and 12 (55%) were employed as nurses. On average, nurses and physicians had 7.5 years (range: 1–23 years) and 6.75 years (range: 4–13 years) of experience treating patients with ARF/RHD, respectively (Table 1).

**Table 1:** Sociodemographic data of participants.


	SITE				

	TOTAL	ARBA MINCH	DEBRE TABOR	DIRE DAWA	GONDAR

Participants (*n*)	22	4	6	4	8

Age (years)	39 ± 10	36 ± 1	38 ± 10	36 ± 9	42 ± 13

Gender [*n* (%)]					

Male	12 (55%)	2	3	1	6

Female	10 (45%)	2	3	3	2

Profession [*n* (%)]					

Nurse	12 (55%)	2	4	3	3

Physician	10 (45%)	2	2	1	5

Experience (years)^†^	7.2 ± 4.8	7.0 ± 4.8	4.8 ± 5.7	8.2 ± 5.3	8.6 ± 3.9


Data are presented as mean ± SD or number (%). ^†^Participant experience (in years) in delivering services for ARF/RHD patients; particularly involvement in assessment and follow up, prescribing antibiotics and injecting BPG for secondary prophylaxis.

Four main themes were identified, with factors influencing the use of BPG for secondary prophylaxis of ARF/RHD characterized as (1) individual factors, (2) health-system related, (3) patient related, or (4) product related. Of the four main themes, 17 subthemes were identified (12 were recognized as barriers and 5 were identified as facilitators) and mapped on TFA and COM-B models (Supplementary Table 5). A summary table of key enablers and barriers of BPG secondary prophylaxis is also provided (Supplementary Table 6).

### Individual factors

All HCPs identified that a significant barrier to the successful delivery of BPG was the fear of a serious adverse event after intramuscular administration. Whilst the majority agreed that BPG was an effective treatment and superior to oral therapy, the availability and willingness of HCPs to prescribe/administer BPG was strongly influenced by concerns about potential adverse reactions. These concerns were further compounded by fears for the HCPs’ own safety, stemming from prior fatalities attributed to BPG administration and anecdotal reports of attempts at retribution by patients’ families against HCPs. This fear was particularly evident in nurses, who are tasked with injecting the medication. Several nurses reported they believed there was a strong correlation between the anticipated incidence of severe adverse events and the health status of the participant on the day of presentation for injection. If the patient was severely unwell, there was a perceived higher risk, compared to patients with mild to moderate disease who presented in reasonable health. Moreover, only a few participants had direct experience with patient deaths following BPG injection, and they noted that all the patients who died after receiving the injection had a history of BPG exposure.

*As experts, we think that if they are seriously ill, they will go into shock due to injection… but we think it will make a difference for those who have mild to moderate RHD*.

(Nurse, Debre Tabor)

… Next to his death, his family said to us we will beat and kill health professionals outside in the city. When they said so, everyone stopped delivering the medication

(Nurse, Gondar)

She comes to us every 2 months and had been on BPG before… She was in critical condition… We administered BPG to her, and then she immediately went into shock… She died while on oxygen.

(Nurse, Debre Tabor)

A strong sentiment was that the ‘…risk of anaphylactic shock scares me’. Ultimately, the fear of severe adverse events occurring post-injection led to a lack of willingness to ‘voluntarily administer the injection’, only doing so because, without the medication, ‘patients suffer’. Whilst this theme was reported across all research sites, in Gondar the fear of anaphylaxis led to few nurses being prepared to deliver BPG, hence less effective oral alternatives were prescribed by doctors at this site. However, most of the nurses did not deny that better treatment outcomes were achieved from BPG administration, compared to oral therapies.

There was also evidence of a level of disconnect between nurses and physicians with respect to liability if severe/fatal adverse events occurred. Nurses indicated that the lack of clinical oversight provided by doctors at the time of injection contributed to their fear. One nurse stated,

*Doctors are not involved other than prescribing. The pressure is on us, and I don’t think doctors understand our worries. There is no doctor you can consult within the injection room. If sudden death happens, it will be considered due to us, nothing else*.

(Nurse, Debre Tabor)

Nurses also perceived that they were the only HCPs at risk (not the prescribing physicians) and would be held responsible if an adverse event occurred after injection. To reduce the burden of responsibility, nurses at several research sites reported they obtained written consent from families/patients prior to BPG as a means of trying to avoid/minimize liability for possible adverse outcomes. At some hospitals (Debre Tabor and Arba Minch), nurses had to be assigned to administer BPG to ensure ongoing service provision.

Physicians highlighted that a significant challenge to the efficient delivery of BPG was the reluctance of hospital nurses to administer intramuscular BPG.

*There is a fear with the nurses, there is a situation where they say that they will not give injections, but they bother us, but in the end, we get along with them*.

(Physician, Dire Dawa)

Physicians also reported that nurses were potentially the most significant barrier to the efficient delivery of BPG, as they are often reluctant to administer the injection. This reluctance was identified as being primarily due to previous experiences with severe adverse events, as well as information they have received about such incidents, threats, and the complexities surrounding the overall context. The low level of patient/community awareness, along with the challenges related to processes and structures (e.g., emergency management of anaphylaxis), has further reinforced the nurses’ reticence in administering BPG.

*Nurses often refuse to inject. It (BPG) is not given in our hospital because they refuse totally. The reason they refuse is because they have experienced death when giving the medicine…because of this, it is heard that they are not giving the medicine and it is spreading to other places, so the information is now becoming a general problem*.

(Physician, Gondar)

Whilst all physicians emphasized the importance of BPG for secondary prophylaxis, and their willingness to prescribe treatment, they acknowledged that the potential danger of anaphylaxis during injection of BPG in patients with advanced RHD was an important consideration.

Regardless of their fears related to drug administration, all physicians and most nurses highlighted the importance of BPG and their perception of clinical improvement in patients who received therapy.

*From the perspective of physicians, there is still better acceptance and confidence on the efficacy of BPG*

(Physician, Gondar)

Moreover, most HCPs perceived that the patients had better treatment adherence with BPG than the alternative oral treatments, with the additional benefit of reduced pill burden.

*We currently only use amoxicillin. The problem with it is the pill burden. Adherence is an issue*.

(Physician, Gondar)

*From what I have read, the efficacy (of amoxicillin) I have seen in the past is equal… adherence is the key. For BPG side effect is the problem whereas for amoxicillin adherence is the problem*.

(Physician, Debre Tabor)

### Health System Realted

A common shared sentiment was that ARF/RHD was a significant health burden in Ethiopia and a leading cause of morbidity and mortality in children. Despite this, health services and disease prioritization did not match that of other diseases.

*As a pediatrician, I would say attention has to be given to the treatment (BPG) like TB and HIV. It is sad to see the children suffer… RHD is a neglected disease*.

(Physician, Arba Minch).

Whilst comprehensive treatment guidelines (14) are readily available in Ethiopia, training and logistics to support and guide BPG administration, and to manage severe adverse reactions, were not readily available at the time of this study. This lack of training and support contrasts with other priority conditions, such as tuberculosis, HIV, and malaria, which may receive more attention due to stakeholder priorities and for which detailed training programs are available to support diagnostic and therapeutic interventions.

Broad health education was highlighted as a significant influencing factor in how the community, and even HCPs, perceived ARF or RHD.

*The community does not understand the sequalae very well. They don’t understand that RHD/heart disease is related to bacteria and the tonsils. So, this needs attention. There is a problem of not understanding the importance of prophylaxis and the disease, even physicians are not close enough to BPG and the prophylaxis*

(Physician, Dire Dawa)

As a result, it was noted that patients often presented with late disease with established complications, and there was a lack of commitment toward treatment compliance. Moreover, several participants noted a suboptimal commitment by some HCPs to immediately commence patients on prophylaxis, adequately monitor treatment compliance, or assess their monthly response to treatment. It was perceived that a lack of understanding of disease pathogenesis and treatment characteristics significantly contributed to the observed behavior of both patients and HCPs.

A further cause for concern was the lack of preparedness and resources that were provided by health centers to support the administration of BPG and respond to associated side effects. Several nurses highlighted that hospitals/injection rooms did not provide adequate supplies of resuscitation kits, adrenaline, medical support, and equipment for monitoring participant health and vital signs.

*The biggest drawback was that when we gave BPG, resuscitation material was not ready. We just take a consent and then inject. We didn’t secure IV early. Adrenaline, mask and other things may not be available. If the patient was shocked, it was not possible to give help on the spot. Although I was not present, the patient expired when they rushed to bring them to ICU*.

*(Nurse, Arba Minch)*

This reported lack of supportive services exacerbated the fear of injecting BPG, particularly in patients who were in end-stage disease or presenting unwell. The availability of BPG was also identified as a limitation. Patients were directed to purchase the drug in private pharmacies when a stable supply was not available in the treating hospital.

*… it was not available for 3 months in the hospital. They brought the medicine from private sources and elsewhere to be injected. There were those who did not get BPG and took amoxicillin alternatively*.

(Nurse, Arba Minch)

Participants also reported that many cases of RHD were in rural areas where health services are not well supported. As a result, patients had to travel great distances, under financial constraints, to attend follow-up appointments in the referral hospital and may not be assessed satisfactorily or in a timely manner. As patients face financial hardship, both for travel and costs of attending hospital/waiting for treatment, this burden was thought to contribute to suboptimal treatment compliance and efficacy, which could be resolved through effective training and additional resources provided in non-referral centers.

*Patients come by appointment, but they come from faraway places, and they are harassed. And it would be good if they were treated locally… When a specialist doctor is needed it is better to come every 2 to 3 months, otherwise they can be treated in their local area… Those who come from far away and spend a lot of money, but only receive medicine and get injected, they are rarely examined during follow-up. If so, they can do some follow-up at their nearest medical facility. For patients, monthly attendance has a significant impact on adherence*.

(Nurse, Dire Dawa)

Co-administration of anesthetic or analgesic agents (lidocaine or diclofenac) also aided compliance during treatment, resulting in less frequent adverse events.

*We do not inject BPG alone. We are giving it with antipain (diclofenac) because the patients go into pain shock easily when we inject them without antipain… if we add antipain, it is not dangerous*.

(Nurse, Debre Tabor)

### Patients/Caregivers Related

It was reported that due to poor health-seeking behavior and limited access to healthcare, many patients were diagnosed with RHD once the disease was well established.

*They come after the patient shows congestive or other non-congestive heart failure symptoms. Therefore, the chances of early detection are slim. At the end they come with severe acute malnutrition and compensated heart failure because they are not seen*.

(Physician, Gondar)

Several HCPs noted that early detection of ARF and prompt corrective treatment could be negated by patients seeking traditional medicines/practices for sore throat in childhood, whereby the patients may undergo uvulectomy at the hands of a traditional healer. The use of unproven traditional treatments (e.g., uvulectomy) and the resulting delay in seeking hospital care increase the potential for disease complications and the missed opportunities for early prevention further driving patient behavior and contributing to poor treatment compliance. Moreover, several participants noted that the over-expectation of prophylaxis among RHD patients (such as expecting it to be curative) and hesitancy to attend monthly follow-ups due to pain, time constraints, and financial concerns all contribute to poor adherence. Feelings of despair after years of follow-up further exacerbate challenges.

*They take BPG more often and they think they will be saved. When there is no change, there is a state of despair and asking what the medicine will do. … as a result, they lose adherence for the follow up*.

(Physician, Arba Minch)

However, strong facilitators for successful treatment were seen as the patient’s/caregivers’ willingness to accept treatment advice provided by HCPs, and their preference for a monthly injection rather than more frequent oral regimen alternatives.

Patients love something that can be injected rather than something that needs to be *swallowed*

(Nurse, Gondar)

### Product related

Both BPG formulation and route of administration were reported to pose significant challenges. The perceived risk of anaphylaxis and/or pain-related cardiac events, thought responsible for the reported cases of death during administration, were also reported to be a significant barrier. However, the pain of injection was linked to the method of administration (deep intramuscular injection) alongside formulation concerns (product brands) resulting in needle blockage. Additionally, factors contributing to needle blockage included delays after reconstitution (due to sedimentation), patient positioning, and administering without local anesthetics or analgesics.

*If you try to inject BPG with delay… it will immediately form a crystal and blocks the needle*

(Nurse, Debre Tabor)

*If you delay, it will dry out in the syringe… the water and the medicine will separate easily, the medicine sediments. If the patient is uncomfortable and then delays you, it will be difficult to push*.

(Nurse, Arba Minch)

*We give it with diclofenac, first it helps to reduce pain and second to further dilute BPG after reconstitution as BPG has crystal forming characteristics*.

(Nurse, Debre Tabor)

*… as I told you, about 6 children died. When we inject BPG, we add about 0.3 cc of lidocaine and inject it mostly on the thigh muscle. A 14-year-old boy died immediately without giving time for resuscitation when one of our friends administered it to him*

Nurse, Gondar

Good nutrition and hydration prior to injection were also reported to prevent or at least minimize adverse events, which was often recommended by nurses to their patients whenever they attended for BPG injection.

## Discussion

Our study is the first to investigate the acceptability and challenges of BPG secondary prophylaxis for ARF/RHD from the perspective of HCPs in multiple centers in Ethiopia, and it is one of few studies worldwide (21,22,40,44). Several factors affecting ARF/RHD secondary prophylaxis have been identified and mapped to the TFA construct, COM-B, and BCW models to elucidate the requirements for behavioral change interventions (42).

Fear of sudden severe adverse reactions, or retribution in the event of fatal reactions, were identified as barriers to consistent and successful BPG implementation. To overcome fear-related barriers, two contrasting scenarios emerged across treatment centers. In centers where BPG delivery continued, many HCPs implemented informal written consent procedures to be signed by family members prior to each BPG injection. Whilst this somewhat alleviated fear of retribution, HCPs perceived that this practice contributed to poor treatment adherence due to increased patient hesitancy to commit to BPG treatment. In contrast, at the University of Gondar Hospital, BPG administration was ceased and replaced by oral amoxicillin, due to the refusal of nursing staff to administer BPG injections. However, decreased treatment adherence, likely due to patient preference for injectables over oral regimens (45,46), was also a perceived outcome. Interestingly, despite a fear of treatment-induced anaphylaxis, providers overwhelmingly perceived BPG as the superior prophylactic treatment for ARF/RHD, which aligns with previous reports (47,48,49). This suggests that the decision not to implement BPG administration in some settings was in part driven by fear of severe adverse events, rather than a lack of confidence in the effectiveness of BPG.

In 2015, Ethiopia hosted the Social Cluster of the African Union Commission to develop a ‘roadmap’ of key actions that needed to be taken by African governments to eliminate ARF and RHD. Seven key priority areas were adopted within this strategy, including decentralizing technical expertise and technology for diagnosing and managing ARF and RHD, initiating national multi-sector RHD programs within noncommunicable disease control programs of affected countries, and ensuring adequate supply of high-quality BPG (23). Despite this, participants in the current study perceived ARF/RHD to be a neglected or low-priority health problem in Ethiopia, likely attributed to an apparent lack of national ARF/RHD control strategy (45) and health system challenges (50). In alignment with the proposed African Union Commission roadmap and RHD control goals established by the WHO (51) and World Heart Federation (7), the organization and strengthening of existing healthcare systems (51,52), defining and adaptation of best clinical practices (5,40,53), and promotion of early detection and treatment are all potential strategies for stronger RHD management programs in high burden developing countries (51). Thus, training HCPs on the burden of RHD, its management, and the risks of BPG injection pain and anaphylaxis, while emphasizing best practices for BPG administration, appears to be essential for improving BPG delivery. Additionally, implementing patient and community awareness programs could enhance the effectiveness of these services, ultimately reducing the burden of RHD and improving health outcomes.

Our study identified that health-seeking behavior of RHD patients, as per the HCPs’ perspective, is poor. It is known that the majority of the Ethiopian population live in rural areas and have low rates of literacy (54) which may contribute to limited access to healthcare services and suboptimal health outcomes (55). By contrast, the use of traditional medicine among the community is significant, with estimates that approximately 80% of the population uses complementary and alternative therapies (56).

As reported in previous studies, the possible risk of severe adverse reaction (57,58,59), pain and needle block are prominent product-related barriers of BPG secondary prophylaxis for ARF or RHD (16,17,33). In this study, HCPs noted that contributing factors for needle blockage and injection pain included delays in administration (after reconstitution, due to sedimentation), patient position (side-lying position), delivering without local anesthetics/analgesics and product brands, as also reported previously (25,53).

Although only a few participants had direct experience with patient deaths following BPG injection, an important observation in our study was that HCPs reported all known BPG administration-related deaths occurred in patients with long histories of receiving BPG as RHD secondary prophylaxis. This suggests that stimulated physiologic cascades (i.e., vasovagal reactions) due to anxiety, poor health, and/or injection pain (24,27,53,60), rather than penicillin anaphylaxis, is the principal driver of fatal outcomes. This finding is consistent with previous reports that fatalities associated with BPG were in patients with severe cardiac compromise (24). Consistent with this concept, the reported prevalence of severe adverse events in patients receiving BPG for syphilis treatment is very low (60,61,62). By comparison, a grey-literature retrospective study of RHD patients in Ethiopia reported a 21% prevalence of BPG-related adverse reactions, the majority of which were limited to injection site swelling, with no fatalities observed (63). These reports underscore the importance of systematic monitoring and reporting of severe adverse reactions following BPG injections. Such practices are essential for generating conclusive pharmacovigilance data, which can inform risk-benefit assessments and evidence-based therapeutic decisions. Auditing treatment outcomes, particularly the incidence and management of severe adverse events associated with BPG, would benefit from the active involvement of HCPs and regulatory bodies to strengthen and fully operationalize adverse reaction monitoring and reporting systems in Ethiopia (64,65) and globally as the safety of BPG intramuscular injection becomes an increasing concern (24,53).

Product-related barriers could be addressed by using alternatives to powdered BPG, such as BPG suspension formulations in pre-filled syringes (e.g., Bicillin^®^). Furthermore, recent reports of subcutaneous delivery of BPG, which are perceived to be ‘less painful and longer-lasting’, may offer a potential solution to the product-related challenges and improve treatment adherence, due to less frequent administration schedules (30,31,32). However, as BPG suspension formulations are more expensive than powdered BPG and require cold-chain storage, deploying BPG suspension in pre-filled syringes to low-income settings would require financial and logistic support from global sponsors and agencies.

Despite the barriers to BPG use for ARF or RHD secondary prophylaxis that were identified in our study sites, important facilitators were reported by participants. For example, HCPs recognized the superior treatment efficacy of BPG for ARF/RHD and improved adherence, compared to alternative oral regimens (45,47,48,49). HCP-initiated techniques to improve treatment delivery were also recognized including the addition of local anesthetics/analgesics for pain relief and prevention of needle blockage, ensuring the patient was placed in a supine position during, and for a period after, BPG injection, and deferring BPG administration in unwell patients (49,53,62). Patient-related facilitators were also identified in this study, including good counseling and a preference for monthly (or less frequent) injections to improve compliance and treatment outcomes (46).

This qualitative study had limitations. For feasibility, study recruitment was limited to hospitals within major urban areas (servicing large catchment areas), with participants approached based on their experience treating RHD. As such, the reported perceptions may not reflect the acceptability, barriers, and facilitators of ARF or RHD secondary prophylaxis of all HCPs across Ethiopia, or in other clinical settings. Another limitation of this study was that it was based on self-reported feedback, which is susceptible to recall bias and may lack a clear distinction, between firsthand experiences and anecdotal accounts. Furthermore, as a single investigator was responsible for data collection, translation, and preliminary analysis, some interpretation/analysis bias may have occurred. However, to mitigate the potential bias, we followed the widely used COREQ checklist (37), an independent review of the translation, the involvement of a second investigator in the extraction and analysis of collected data, and contributions from all investigators in the interpretation and reporting of our results.

## Conclusion

Our study revealed that although BPG is the mainstay of ARF and RHD therapy in Ethiopia, several barriers impact successful service delivery and patient outcomes. Despite a low incidence of BPG injection-related severe adverse events, anecdotal ‘historical evidence’ appeared to drive HCP anxiety with respect to BPG administration and impending successful BPG implementation. We suggest that improved resourcing of RHD services would be highly beneficial, including comprehensive education of HCPs on RHD prevention, management, and risk mitigation as well as training in BPG administration and management of critical incidents (e.g., anaphylaxis), and the provision of essential resuscitation equipment. Enhancing patient and community education to improve awareness of RHD and perceptions of therapy-related adverse outcomes also could address the current barriers and shift the paradigm of poor BPG acceptability. Finally, in addition to considering improved BPG formulations, pharmacovigilance research on risks of severe adverse reactions to BPG and identifying associated risk factors, would provide valuable evidence to support RHD secondary prophylaxis and inform the management of potential adverse reactions.

## Additional Files

The additional files for this article can be found as follows:

10.5334/gh.1393.s1 Supplementary Table 1. COREQ 32-item checklist.

10.5334/gh.1393.s2 Supplementary text File. Interview topic guide.

10.5334/gh.1393.s3 Supplementary Table 2. COM-B model definition.

10.5334/gh.1393.s4 Supplementary Table 3. TFA constructs definition.

10.5334/gh.1393.s5 Supplementary Table 4. BCW definition.

10.5334/gh.1393.s6 Supplementary Table 5. Themes mapped on TFA and COM-B.

10.5334/gh.1393.s7 Supplementary Table 6. Key enablers and barriers to BPG secondary prophylaxis.
