# Supplementary material for: Acceptability and Implementation Challenges of Benzathine Penicillin G Secondary Prophylaxis for Rheumatic Heart Disease in Ethiopia: A Qualitative Study
Source: Glob Heart. 2025 Jan 29;20(1):8. doi: 10.5334/gh.1393 (PMC11784522; doi:10.5334/gh.1393)
Supplement: Supplementary text File. — Interview topic guide. [file gh-20-1-1393-s2.pdf]

Interview topic guide

1. How long have you been providing care for ARF/RHD cases, and what are your thoughts on this experience?
2. Are there any concerns you have regarding the management or care of ARF/RHD patients using secondary prophylactic agents?
3. How do you perceive the acceptability of BPG secondary prophylaxis among:
    a. health care providers,
    b. RHD patients and families of ARF/RHD cases?
4. What are the challenges, facilitators, and barriers related to the implementation of BPG secondary prophylaxis for ARF/RHD? Discuss from different perspectives?
5. Have you ever used other prophylactic agents (e.g., for prescribing or delivering), and what are your views on them? Please discuss reasons for preference, limitations, and other relevant factors.
6. What actions are needed to improve the management of ARF/RHD?
7. Do you have any final comments or questions?
