# Supplementary material for: Acceptability and Implementation Challenges of Benzathine Penicillin G Secondary Prophylaxis for Rheumatic Heart Disease in Ethiopia: A Qualitative Study
Source: Glob Heart. 2025 Jan 29;20(1):8. doi: 10.5334/gh.1393 (PMC11784522; doi:10.5334/gh.1393)
Supplement: Supplementary Table 2. — COM-B model definition. [file gh-20-1-1393-s3.pdf]

Table 2: The Capability, Opportunity, Motivation and Behaviour (COM-B) model's domains and their definitions

COM-B model domains	Definitions
Capability (psychological and physical	Individual person's psychological and physical capacity to engage and perform the desired activity or behaviour. This includes having the required necessary knowledge and skills.
Opportunity (social and physical)	All external factors in person's environment or circumstances that encourage or discourage the desired behaviour. This includes time, resource, and norms of practice.
Motivation (reflective and autonomic)	A coherent set of brain process that determines person's displayed personal qualities in a social and work setting. This includes habitual processes, emotional responding, analytical decision making and professional confidence.

Adopted from Michie S, van Stralen MM, West R. The behaviour change wheel: a new method for characterising and designing behaviour change interventions. Implement Sci 2011; 6:42–53.
