# Supplementary material for: Acceptability and Implementation Challenges of Benzathine Penicillin G Secondary Prophylaxis for Rheumatic Heart Disease in Ethiopia: A Qualitative Study
Source: Glob Heart. 2025 Jan 29;20(1):8. doi: 10.5334/gh.1393 (PMC11784522; doi:10.5334/gh.1393)
Supplement: Supplementary Table 3. — TFA constructs definition. [file gh-20-1-1393-s4.pdf]

Table 3: Definitions of the component constructs in the Theoretical Framework of Acceptability (TFA)

**TFA Construct**	**Domain**
Affective attitude	How an individual feels about the intervention
Burden	The perceived amount of effort that is required to participate in the intervention
Ethicality	The extent to which the intervention has good fit with an individual's value system
Intervention coherence	The extent to which the participant understands the intervention and how it works
Opportunity costs	The extent to which benefits, profits, or values must be given up engaging in the intervention
Perceived effectiveness	The extent to which the intervention is perceived to be likely to achieve its purpose
Self-efficacy	The participant's confidence that they can perform the behaviour(s) required to participate in the intervention

Adopted from: Sekhon M., Cartwright M, & Francis JJ. Acceptability of healthcare interventions: An overview of reviews and development of a theoretical framework. BMC Health Services Research 2017; 17:88
