# Supplementary material for: Acceptability and Implementation Challenges of Benzathine Penicillin G Secondary Prophylaxis for Rheumatic Heart Disease in Ethiopia: A Qualitative Study
Source: Glob Heart. 2025 Jan 29;20(1):8. doi: 10.5334/gh.1393 (PMC11784522; doi:10.5334/gh.1393)
Supplement: Supplementary Table 4. — BCW definition. [file gh-20-1-1393-s5.pdf]

Table 4: Definitions of interventions and policies within the Behaviour Change Wheel

Interventions	Definition	Examples
Education	Increasing knowledge or understanding	Providing information on the prevention and management of ARF/RHD through campaigns, schools, hospitals
Persuasion	Using communication to induce positive or negative feelings or stimulate action	Using messaging to encourage regular follow-up appointments for secondary prophylaxis in RHD patients.
Incentivisation	Incentivisation Creating expectation of reward	Offering rewards or incentives for adherence to RHD medication schedules and follow-up care.
Coercion	Creating expectation of punishment or cost	Implementing policies that require RHD patients to attend regular check-ups or face penalties.
Training	Imparting skills	Training healthcare providers on the correct administration of BPG and management of adverse reactions.
Restriction	Using rules to reduce the opportunity to engage in the target behaviour (or to increase the target behaviour by reducing the opportunity to engage in competing behaviours)	Restricting the sale of over-the-counter medications that may interfere with RHD treatment.
Environmental restructuring modelling	Changing the physical or social context	Providing healthcare infrastructure to support regular BPG administration in rural/remote areas.
Modelling	Providing an example for people to aspire to or imitate	Using media to showcase individuals adhering to RHD prevention guidelines, encouraging others to follow
Enablement	Increasing means/reducing barriers to increase capability or opportunity	Providing mobile health services to improve access to RHD secondary prophylaxis in underserved areas.
**Policies**		
Communication/ marketing	Using print, electronic, telephonic or broadcast media	Conducting broadcast media campaigns, using print, electronic, telephonic
Guidelines	Creating documents that recommend or mandate practice. This includes all changes to service provision	Producing and disseminating treatment protocols
Fiscal	Using the tax system to reduce or increase the financial cost	Incentivize behaviour, allocating government funding for RHD prevention programs
Regulation	Establishing rules or principles of behaviour or practice	Establishing rules or principles of behaviour or practice to guide clinical practice
Legislation	Making or changing laws	Enacting laws that mandate screening for rheumatic fever in schools or requiring health insurance to cover RHD prevention and treatment.

Environmental/ social planning	Designing and/or controlling the physical or social environment	Integrating RHD prevention into community health programs and ensuring access to healthcare in underserved areas.
Service provision	Delivering a service	Establishing community-based RHD prevention programs, including outreach to schools and clinics for early detection and management of rheumatic fever.

*Adopted from Michie S, Van Stralen MM, West R. The behaviour change wheel: a new method for characterising and designing behaviour change interventions. Implementation Science 2011, 6(1):42-53
