# Supplementary material for: Acceptability and Implementation Challenges of Benzathine Penicillin G Secondary Prophylaxis for Rheumatic Heart Disease in Ethiopia: A Qualitative Study
Source: Glob Heart. 2025 Jan 29;20(1):8. doi: 10.5334/gh.1393 (PMC11784522; doi:10.5334/gh.1393)
Supplement: Supplementary Table 5. — Themes mapped on TFA and COM-B. [file gh-20-1-1393-s6.pdf]

Table 5: Identified themes mapped on the domains of Theoretical Framework of Acceptability (TFA) and COM-B and BCW framework

Factors influencing BPG secondary prophylaxis for ARF/RHD		COMB-B domain	TFA construct	Sample quotes from interviewees	Solution taken and/or suggested	Intervention and policy functions of BCW
Themes	Sub-themes					
Health care provider related	Fear of anaphylaxis	Motivation	Opportunity cost	*"And every time when a patient with BPG injection comes, we(nurses) say 'ohh God forgive us please'. Even when we finish our duty of the injection room after a year, we cheer and congratulate each other"* *52-year-old Nurse, Debre Tabor*	Research and Training	Training and Enablement intervention: Communication category
	Reluctance to deliver BPG	Motivation	Affective attitude	*"The hospital tried to force them (i.e., nurses), and they said take the associated risk. Who will take the risk? An attendant who comes with a patient can fight the health care provider when there is death. What do you take then, the medicolegal case can be taken by the hospital. But when an incident happens, let alone about another person, you don't even know about yourself. Who will take the responsibility?"* *Gondar, 42 Years old Physician*	Public awareness and counselling, Availability and resourcing of emergency medical treatment in place Taking consent	Education and persuasion: Communication category
	Perceived treatment outcomes	Motivational	Perceived effectiveness	*"I would love for BPG not to disappear. However, if there is a better substitute it would be good to avoid the injection pain. In many patients we have seen a good outcome, and thus, the drug is very good, it makes a difference"* *Debre Tabor, 33 years old Nurse*	Research better to be done further. E.g., breakthrough prevalence/recurrence, Safety	
	Learning from and adopting good BPG injection experience	Motivation	Intervention coherence	*"However, for those who come from urban areas and are legal experts, they should sign a consent. The consent is not a legal or institutional form, we offer them to sign if in case something goes wrong for evidence"* *Gondar, 63 years old Nurse*	Use of anesthetics or analgesics, Defer BPG injection if the patient is unwell or has vital symptoms on day of BPG injection, Consenting	Education, Training: communication category
	Neglected/unprioritized health problem	Opportunity	Affective attitude	*"Although I don't have study reports, for me the RHD burden is comparable with TB/HIV, even if it is not that much it is significant. Thus, it needs policy attention and training, which will make professionals, and the people close to the disease and its prevention"* *Dire Dawa, 29 years old Physician*	Health policy should give attention for this major public health problem	Education, Training, Enablement: Communication and guidelines category

Health system related	Lack of public and health care providers awareness and training	Capability	Affective attitude	*"The community does not understand the sequalae very well. They don't understand that RHD/heart disease is related to the bacteria and the tonsils. So, this needs attention. There is a problem of not understanding the importance of prophylaxis and disease, even physicians are not close enough to BPG and the prophylaxis"* *Dire Dawa, 29 years old Physician*	Health education and public awareness via different media and	Enablement, Education, Training intervention: communication category
	Lack access or availability of resources	Opportunity	N/A	*"Sometimes the medicine wears off. Six months ago, it was not available for 3 months in the hospital. They brought the medicine from private sources and elsewhere to be injected. There were those who did not get BPG and took amoxicillin alternatively"* *Arba Minch, 36 years old Nurse*	Improving the supply of drugs and medical supplies, supporting patients	Enablement: Regulation category
	Recommendation of BPG as a first line agent for RHD prophylaxis (guidelines available) and coadministration of local anesthetics/analgesics	Opportunity	Intervention coherence	*"In fact, there is a guideline to prescribe BPG even for tonsillopharyngitis so that BPG can be adapted. The system encourages to issue BPG. But the problem is to go down to the bottom and convince the experts to administer it"* *Gondar, 42 Years old physician*	Training and further guidance on how to promote BPG delivery required	Education, Training, Enablement: Communication and guidelines category
Patients or caregivers related	Over expectation and despair	Opportunity	Perceived effectiveness	*"They take BPG more often and they think they will be saved. When there is no change, there is a state of despair and asking what the medicine will do. The caregivers say he/she has breathing difficulties and gets tired, and as a result they lose adherence for the follow up"* *Arba Minch, 38 years old Physician*	Functioning local health institutions, Support for health care cost, when necessary, Health education	Enablement, Environmental restructuring: Environmental planning and Education category
	Poor health seeking behavior	Opportunity	N/A	*"In our setup (in Africa) patients come after repeated attacks. Not all RHDs cause heart disease, has a limited proportion. However, when it is repeated, the probability of causing heart disease is high, and the severity increases"* *Arba Minch, 37 years old Physician*	Public awareness, outreach campaign	Education: communication category
	Good acceptance of HCPs counselling	Opportunity	Intervention coherence	*"Of course, they will have some panic when you tell patients about the possible risk associated with the medication. However, as far as I know, there was no patient to the extent of refusing the medication, it is rare. The problem is that health care providers don't give, deliver it"* *Gondar, 42 years old Physician*	Awareness at community/society level is recommended	Education; Communication category

	Traditional medicine utilization	Opportunity	N/A	*"When there is a sore throat, most parents say 'ankar wordot' and what they do is traditional tonsillectomy which is not actually tonsillectomy, rather than treating it with antibiotics"* *Gondar, 63 years old Nurse*	Health education and public awareness	Education: Communication category
	Injectable medication preference	Opportunity	Affective Attitude	*"It is well received by patients. In fact, patients prefer injections rather than swallowing. Generally, the people of our country like/accept injections"* *Debre Tabor, 49 years old nurse*		
	Attempt of retribution for adverse outcomes following BPG injection	Opportunity	Opportunity cost	*"Because of this, as we have our children, we started thinking about ourselves and wrote a letter to the hospital. Next to his death, his family said to us we will beat and kill health professionals outside in the city. When they said so, everyone stopped delivering the medication"* *Gondar, 39 years old Nurse*	Written consent and legal protection HCPs	Education: Communication and regulation category
Product related	Shock/sudden death following injection	Opportunity	Opportunity cost	*"… as I told you, about 6 children died. When we inject BPG, we add about 0.3 cc of lidocaine and inject it mostly on the thigh muscle. A 14-year-old boy died immediately without giving time for resuscitation when one of our friends administered it to him"* *Gondar, 63 years old Nurse*	Checking patient's vital symptoms, adequate food, and fluid intake.<br>Injecting in lay down/supine position<br>Further study on the drug	Guidelines policy category
	Needle block	Opportunity	Opportunity cost	*"In my experience, soon after reconstitution, it must be given immediately. The water and the medicine will separate easily, the medicine sediments. If the patient is uncomfortable and then delays you, it will be difficult to push. I must change one or more needles"* *Arba Minch, 36 years old Nurse*	Changing needle and injecting immediately after reconstitution<br>Adding other drugs PPF, diclofenac, and lidocaine<br>Using large syringes	Guidelines policy category
	Pain of injection	Opportunity	Opportunity cost	*"It hurts a lot. They tell us that it has a burning sensation. I don't know the scientific thing, but we give a little lidocaine on the tip"* *Arba Minch, 36 years old Nurse*	Lidocaine, or diclofenac is commonly used	Guidelines policy category

BCW: Behavioural Change Wheel, COM-B: Capability, Opportunity, and Motivation-Behaviour, TFA: Theoretical Framework of Acceptability, PPF: Procaine penicillin fortified; HCP: Health care professional
