# Supplementary material for: Acceptability and Implementation Challenges of Benzathine Penicillin G Secondary Prophylaxis for Rheumatic Heart Disease in Ethiopia: A Qualitative Study
Source: Glob Heart. 2025 Jan 29;20(1):8. doi: 10.5334/gh.1393 (PMC11784522; doi:10.5334/gh.1393)
Supplement: Supplementary Table 6. — Key enablers and barriers to BPG secondary prophylaxis. [file gh-20-1-1393-s7.pdf]

Tabel 6. Summary of key enablers and barriers to BPG secondary prophylaxis as described by HCPs at tertiary care facilities in Ethiopia.

**Theme**	**Enablers**	**Barriers**
Individual factors	• Knowledgeable of treatment options (physicians)<br>• Acknowledge treatment efficacy of BPG.<br>• Learning and/or adopting good experience in BPG administration	• Often nurses solely responsible for delivering injections and are more often not willing to do so.<br>• Fear of severe adverse event occurring whilst delivering medication (nurses) because of previous mortality cases in children and threats from family.<br>• Lack of training and education for those delivering therapy.<br>• Disconnect between different levels of HCPs during BPG injections and perceived differences in liability in the event of severe adverse reactions.<br>• Avoiding BPG injection and patients need to take oral alternatives
Health system related	• Recommendation of BPG as a first line agent for RHD prophylaxis (guidelines available)	• BPG stock outs, and often when available in only major or zonal hospitals, not at site where most patients reside.<br>• Injection rooms are not well resourced with appropriate resuscitation equipment or physician support (for severe cases)<br>• Gaps in staff training on disease diagnosis, treatment, and recommended approaches of RHD treatment with BPG<br>• Community awareness on RHD prevention and benefits of adherence to the treatment is poor, especially rural dwellers.<br>• Longer waiting time and inadequacy of follow up medical check-ups.<br>• Lack of RHD registry-based treatment

		• Absence of support systems for patients from socioeconomically poor background and who lost follow up
Patient related	• Positive attitude towards HCP treatment advice<br>• Good reliance or acceptability on injectable medications	• Over expectation of RHD prophylaxis (such as curative outcome) and hesitancy for the monthly follow up due to pain, time and financial reasons followed by despair, and poor adherence.
Product related	• Use of local anaesthetic/analgesic agents provided pain support and ease of delivery.<br>• Recognized/acknowledged efficacy	• Intramuscular injection is painful, and the required slow delivery of large drug volumes can lead to severe adverse events (pain shock, stress, anaphylaxis).<br>• Reconstituted BPG can crystalise or sediment quickly, leading to needle blockage.
